# Investigating impacts from topsoil stockpile height on soil microbial communities

**DOI:** 10.3389/frmbi.2025.1607677

**Published:** 2025-09-18

**Authors:** Ashley Fischer, Jay P. Singh, Jonathan Van Hamme, Eric Bottos, Lauchlan H. Fraser

**Affiliations:** Thompson Rivers University, Kamloops, BC, Canada

**Keywords:** mine reclamation, soil microbial ecology, ecosystem restoration, soil response to disturbance, high-throughput sequencing

## Abstract

Mining activities are often severely disruptive to the landscape, and a significant barrier to reclamation following mining operations is the lack of quality topsoil. This project addresses knowledge gaps in the industry by exploring the compositional nature of topsoil stockpiles and their ability to facilitate post-mining revegetation after long-term storage. To do this, we conducted a microbial profiling of two topsoil stockpiles in the interior of British Columbia, Canada. Both stockpiles show depleted soil quality and significant changes compared to reference soils. Notably, there were declines in microbial diversity and significant shifts in community structure with increasing stockpile depths in one of the stockpiles. These results highlight the influence of topsoil-stockpile height on microbial communities in the soil, which ultimately influences the success of restoration. This research can help the industry to optimize restoration and expedite recovery in their mine-closure practices and provides insights into the general structure of the microbiome existing across a gradient in severely disturbed mining soils.

## Introduction

The mining industry in British Columbia is an essential source of resources and is a key contributor to the economy. As of 2020, there were 16 major metal and coal mines, and during 2020, the mining industry produced approximately $7.3 billion and provided more than 30,000 jobs (Ministry of Energy, Mines and Low Carbon Innovation, 2020) mining activities typically include mineral exploration, mineral development, mine production, and mineral processing. These disturbance events cause lasting negative environmental impacts such as ecosystem degradation, habitat destruction, pollution, and loss of soil carbon (Tripathi et al., 2016). Federal and provincial regulations ensure that the restoration or reclamation of landscapes is conducted at mine sites to repair disturbances and return the land to a sustainable ecosystem with a historical level of productivity. Mining is particularly damaging to ecosystems because soils are stripped from landscapes and require reconstruction, which takes hundreds or thousands of years if left to natural processes (Bradshaw, 1997).

Topsoil is a valuable bioactive substrate hosting a wide variety of organisms, including plants, microorganisms, animals, viruses, and protists. Specifically, topsoil is defined as the uppermost part of the soil profile, typically ranging in depth from 7 to 25 cm, and contains the highest concentration of organic matter and microorganisms (Darmody et al., 2009). Preserving topsoil is valuable for mining operations because it could be used later for ecosystem rehabilitation. As such, stockpiles of topsoil are stored for future use. Stockpiling topsoil can be used post-mining to provide nutrients, structure, seeds, and to amend waste materials on site. However, long-term storage of topsoil has been shown to deteriorate soil health by altering its geochemical properties and microbial communities (Ghose and Kundu, 2004; Gorzelak et al., 2020; Harris et al., 1989; Mummey et al., 2002). For example, Boyer et al. (2011) observed that topsoil stockpiles contained compacted and anaerobic soil below one meter, resulting in a low abundance and diversity of earthworms. Moreover, a laboratory study found that stockpiling topsoil caused a decrease in soil organic matter degradation rate, implying diminished microbial activity (Felton and Taraba, 1994).

Soil health is key in restoration success and ecosystem functioning; soil organic matter, soil nutrients, and soil microorganisms play major roles in maintaining healthy, sustainable ecosystems. Currently, only a small proportion of microbial diversity has been identified (Rodrigues et al., 2025). Soil microorganisms provide many ecosystem functions, including roles as biological regulators, chemical cyclers, and ecosystem engineers (Saccá et al., 2017), but are extremely sensitive to disturbances and may take decades for recovery (Bastida et al., 2008; Mummey et al., 2002). Therefore, soil microorganisms are a key component to steering the recovery of disturbed ecosystems, and research on soil microbial functions and communities in various ecosystems and scales is essential.

Special attention should be paid to soil microbial communities in the revegetation of mine soil, where severe, large-scale disturbance occurs, particularly because soil microbes are large drivers in organic matter decomposition and nutrient cycling of macro- and micronutrients critical for plant growth and establishment (Gruber, 2015). Furthermore, soil bacteria and fungi form close relationships with plants, providing services such as nutrient acquisition and protection against environmental stress and pathogens. For example, Chen et al. (2007) found that mycorrhizal colonization significantly increased plant growth in copper (Cu) contaminated soils. This was thought to be a result of increased phosphorus (P) acquisition and decreased Cu concentrations in plant roots. Using their extended, root-like hyphae network, arbuscular mycorrhizal fungi (AMF) can acquire P in areas that are difficult to access. Additionally, AM fungi secrete organic acids, activating unavailable P sources, and can increase the expression of phosphate transporter genes in plants (Wang et al., 2017). Therefore, soil bacteria and fungi are paramount to the restoration process of a landscape and to maintaining a sustainable and resilient ecosystem. The inclusion of microbial community composition data when monitoring soil quality provides crucial insights and a more accurate understanding of how land management impacts the soil ecosystem.

Microorganisms cultured in the lab for study and characterization have been occurring since the 1870s. However, the use of sequencing in the 2000s highlighted that the number of observable, culturable bacteria and fungi are just a tiny fraction of those that are present but unseen (Nilsson et al., 2019). Therefore, molecular techniques, including high-throughput DNA sequencing, have been used as an alternative to culturing and characterization of microorganisms. Characterizing soil microbial communities in mining operations has the potential to aid our ability to restore degraded sites by providing insights and improving our understanding of how land management impacts the soil ecosystem, determining the responses of soils to disturbances, and assessing ecosystem sustainability.

Understanding how microbial communities respond to disturbance and how they recover is critical for optimizing restoration practices. Additionally, at a time when high-throughput sequencing technology has provided novel information about microbial taxa, we can characterize the microbial communities throughout the topsoil stockpile depths, which will provide new information to the field of ecological restoration. Although it is well understood that soil microbial communities are essential for many ecosystem functions, it is less well known how soil microbial activities and composition are impacted by mining disturbance. There is a growing body of literature that has examined the impact of stockpiling on microbial communities. Harris et al. (1989) found that a reduction in aerobic bacteria and biomass with increasing stockpile depth. Ezeokoli et al. (2019) report a decline in soil enzymatic activity and population of beneficial microbial taxa with depth. Similarly, Cabrera-Hernandez et al. (2025) and Hernandez et al. (2024) reveal a shift in bacterial and fungal community composition in stockpiles compared to reference sites. While these studies provide an insight into microbial communities in the shallower stockpiles, very little is known about microbial communities in deeper stockpiles (> 300 cm). Examining soil microbial community composition across a known environmental gradient from the top to the bottom of topsoil stockpiles has the potential to provide insights into factors that shape the microbiome in stored soils and address knowledge gaps. This study aims to improve our understanding of how bacterial and fungal communities in topsoil respond to soil disturbance from stripping, piling, and long-term storage on mine sites by providing soil microbial community composition across a depth gradient. Research investigating soil responses over varying disturbance events and across a range of ecosystem types is critical to developing a unifying theory of ecosystem resilience and recovery. This is especially critical at a time when anthropogenic forces, including climate change, are causing increasingly extreme changes to the environment.

## Methods

### Study sites

#### New Afton Mine

New Gold’s New Afton copper-gold mine (50.654442, −120.509320) was located approximately 10 kilometers west of Kamloops in British Columbia’s Southern Interior. It is situated within the historic Afton Mine, formerly owned by Afton operating Corp. New Afton began commercial production in July 2012 and is the largest underground hardrock mine in Canada. It comprises of underground workings, historic support facilities, a historic open pit, a concentrator, and a tailings facility. The end land use objective is to return the ecosystem to native grasslands that support wildlife and traditional hunting opportunities by First Nations. New Afton is located within the traditional territories of the Tk’emlúps and Skeetchestn Bands. These bands are part of the larger cultural group known as the Secwépemc or Shuswap First Nation. Additionally, New Afton is in the Bunchgrass (BGxw1) biogeoclimatic zone at approximately 700 m in elevation. The Biogeoclimatic Ecosystem Classification (BEC) system in British Columbia incorporates information on climate, soils, and vegetation to provide a framework for management practices (Meidinger and Pojar, 1991). The BGxw1, commonly known as the “middle grasslands”, is dominated by bluebunch wheatgrass, Junegrass, big sagebrush, and rabbit brush (Lloyd et al., 1991). The primary soil orders within the mine site include Tranquille, Trapp Lake, Godey, and Timber. These soils are moderately saline and calcareous and are dominated by Orthic Brown and Dark Brown Chernozems with one occurrence of an Eluviated Eutric Brunisol soil (Government of British Columbia, 2018). The tailings in the New Afton mine are alkaline (pH >8.5) and are high in copper and molybdenum.

#### QR mill

Barkerville Gold Mines Ltd. (BGM) (formally International Wayside Gold Mines) (52.670306, −121.783556) is a Canadian company headquartered in Toronto, Ontario. Barkerville Gold Mines Ltd. BGM has various gold mines around the Barkerville-Wells area. Barkerville Gold Mines’ Quesnel River (QR) mill in Cariboo is located approximately 80 km east of the city of Quesnel in the southern interior of B.C. Ore, concentrate, and waste rock from the Bonanza Ledge Mine and Cariboo Gold Mine are transported, stored, and processed in the QR mill. The current reclamation goals set for QR mill are to restore the landscape so that it does not require further human intervention. QR mill is situated in the traditional territories of the Secwépemc or Shuswap First Nation and lies within the moist, warm Sub-Boreal Spruce (SBSmw) BEC zone based on the BC provincial BEC system. The SBSmw is part of the Canadian Boreal Forest Region and Quesnel Highland (mean annual precipitation ranges from 440–900 mm) and is dominated by Douglas-fir, red-stemmed feathermoss, knights plume, hybrid white spruce, subalpine fir, and electrified cat’s-tail moss (Annas and Coupé, 1979). The QR mill and surrounding area are dominated by Bedenesti, Deserters, and Dominion soil orders. These soils consist primarily of Brunisolic Gray Luvisols and Luvisolic Humo-Ferric Podzols that were deposited by glacial ice (Government of British Columbia, 2018).

### Soil sampling

#### New Afton Mine

New Afton has a 6-year-old, 25-meter-deep topsoil stockpile with approximately 250,600 m^3^ of topsoil materials. Because additional topsoil materials have been added throughout the mine life, the oldest soil is at the bottom of the stockpile, and the youngest soil is at the surface. Four soil cores were extracted via solid stem auger drilling by Geotech Drilling Ltd, provided by New Afton. during September 26th and 27th of 2018, with each core being approximately 3 meters apart. The first 1.53 m was sampled in 0.3 m increments, then at 0.3 m intervals until the bottom at 13.7 m. Thus, sampling depths were at 0.3 m, 0.6 m, 0.9 m, 1.2 m, 1.5 m, 3.0 m, 4.6 m, 6.1 m, 7.6 m, 9.1 m, 10.7 m, 12.2 m, and 13.7 m. The outer 1 cm of the soil core was discarded to ensure that the upper layers did not contaminate the collected soil. Soil was placed into two 1 L Whirl-Pak^®^ bags and Falcon^®^ tubes as they were pulled up soil from the stockpile. Post-collection, samples were combined by depth intervals of the stockpile as follows: 0.0–0.6 m, 0.61–1.5 m, 1.5–6.1 m, and 6.11–13.72 m. The soil stockpile at the time of sampling was sparsely vegetated with grasses and weedy species, likely from natural regeneration. A nearby grassland site was sampled as a reference site, where approximately 6 kg of soil from the top 10 cm was collected using a trowel. The Whirl-Pak^®^ samples were stored in a −20°C freezer at the Research Greenhouse, and the Falcon^®^ tube samples were stored in a −80°C freezer in the TRUGen laboratory at Thompson Rivers University (TRU) until analysis.

#### QR mill

QR mill has a 20-year-old, 6-meter-deep topsoil stockpile. It is a combination of organic soil and general till soil stripped from the surface layers. The stockpile was intended to cover and re-contour during post-mining reclamation. Sampling at the QR mill of Barkerville Gold Mines Ltd. was completed in May 2019. Three soil pits approximately 100 m apart were dug using an excavator in May 2019 to access various layers of the stockpile from the surface to a depth of 575 cm. In the field, soil was placed into two 1 L Whirl-Pak^®^ bags and Falcon^®^ tubes as soil was removed from the stockpile. The stockpile at the time of sampling had vegetation cover, including cottonwood stands in the northwest corner. An adjacent undisturbed forested site was sampled as a reference site, where approximately 16 kg of soil from the top 10 cm was collected. The soil samples at QR mill were collected at different depths than New Afton due to operational constraints. As such, the samples were lumped differently. At QR mill, the samples were aggregated in groups: 0–0.6 m, 0.75–2.6 m, 3.5–3.9 m, and 5.0–5.75 m. After collections, the Whirl-Pak^®^ samples were stored in a −20°C freezer at the TRU Research Greenhouse, and the Falcon^®^ tube samples were stored in a −80°C freezer in the TRUGen laboratory at TRU until analysis.

### Soil geochemistry

Soil samples were prepared for geochemical analyses by drying them at 70°C for 24 hours to constant weight. The samples were then sieved using a 1 mm sieve and ground to a fine powder using a mortar and pestle. The sieved samples were then sent to the Analytical Laboratory at the Ministry of Environment and Climate Change Strategy in Victoria, B.C., for analysis. The total soil Al, B, Ca, Cu, Fe, Mg, Mn, P, K, S, and Zn were determined using inductively coupled plasma–optical emission spectrometry (ICP-OES) after microwave digestion in an acid mixture (Sandroni, 2003). Available P was measured after extraction via Bray P-1 extraction and UV spectrophotometry (Bray and Kurtz, 1945). Available NH_4_-N and NO_3_-N were quantified in an autoanalyzer after extraction in 1M potassium chloride (Kachurina et al., 2000). Organic matter was measured in-house via loss-on-ignition (Singh et al., 2019b). Loss-on-ignition was calculated by weighing approximately 1.5 g of soil into pre-weighed tins and then heating at 500°C for 5 hours. pH and EC were determined using a Palintest^®^ 800 meter. Total Cand N amounts were measured in-house with a ThermoScientific CHNS Elemental Analyzer.

### DNA extraction and sequencing

The soil microbial community composition in each sample was characterized for both fungal and bacterial OTUs in the Applied Genomics Laboratory at TRU. Deoxyribose nucleic acids (DNA) from the soil samples were extracted using the MagAttract PowerSoil DNA Kit (Qiagen Inc.). Extractions were followed by DNA quantification using Qubit™ dsDNA HS Assay Kit (Thermo Scientific). The set of primers: 341F and 806R, was used to amplify the 16S rRNA gene (Ramirez et al., 2017), and the set of primers: ITS86F and ITS4R were used to amplify the internal transcribed spacer (ITS) region between the 5.86S rRNA gene and the 28S rRNA genes (Vancov and Keen, 2009). Samples were run through a second round of polymerase chain reaction (PCR), including the addition of barcoded primers. The thermocycler program for the first round bacterial and fungal amplicon generation consisted of: 95°C for 4 min followed by 25 cycles of 95°C for 30 seconds, 53.4°C for 45 seconds, and 72°C for 2 minutes, with a final extension at 72°C for 5 minutes. For generating sequencing barcode-adapted bacterial and fungal amplicons, the thermocycler program was the same except that 20 cycles were used with an annealing temperature of 65°C.

After the first and second rounds of PCR, the amplicons were purified using the Inovant and AgenCourt AMPure (Beckman Coulter Inc.) magnetic beads before sequencing. Barcoded amplicons were quantified using a Qubit dsDNA HS Assay Kit (Thermo Fisher Scientific, Mississauga, ON) and visualized on an agarose gel. The samples were extracted from the agarose gel using the E.Z.N.A Gel Extraction Kit (Omega Bio-Tek) and were pooled together in equimolar amounts. After pooling, the samples were sequenced on an Ion S5XL (ThermoFisher Scientific).

### Sequence processing and statistical analysis

Raw sequences were demultiplexed in AMPtk 1.5.5 using the amptk ion script with default settings and –trim-len set to 350 bases. Demultiplexed data were imported into Qiime 2 with the qiime tools import script set with –input-format SingleEndFastqManifestPhred33V2 (Bolyen et al., 2019). Denoising was done with DADA2 denoise-single with max-ee set to 1.0 (p-trunc-len set to 0 because the reads were previously trimmed in AMPtk) (Callahan et al., 2016). The Greengenes2 database (version 2022.10) (McDonald et al., 2024) was used to classify the 16S rRNA gene sequences for taxonomic classification. For fungi, version 9 of the Qiime2-compatible UNITE reference database was used to classify the ITS sequences. After processing the sequencing data, any samples with fewer than 1000 reads were dropped. Rare ASVs (read less than 0.005%) were also dropped from the count table. After dropping low read samples and rare ASVs, New Afton had 1675 bacterial and archaeal ASVs and 3166 fungal ASVs. QR mills had 1478 bacterial and archaeal ASVs and 2565 fungal ASVs. Further, we built phylogenetic trees for 16S and ITS representative sequences for downstream analysis. The representative ASV sequences (both 16S and ITS) were first aligned with Mafft (version 7) (Katoh and Standley, 2013), and a maximum likelihood tree was generated using FastTree 2 (Price et al., 2010). The downstream statistical analyses for 16S and ITS data were the same for both sites and are outlined next. Species richness (SR) for 16S and ITS sequences for New Afton and QR mill was measured, and Faith’s phylogenetic diversity (PD) was measured using the count data and the phylogenetic trees. PD and SR of the samples were estimated using the picante R package (version 1.8.2) (Kembel et al., 2010). A linear regression model was used to examine the impact of stockpile depth on alpha-diversity (i.e., PD and SR). A *post hoc* multiple comparison was also conducted using the Benjamini-Hochberg correction to test the significance between stockpile depths. Moreover, the relative abundance of the most abundant phylum was visualized with a stacked bar plot. Phyla with relative proportions over 1% were shown, while phyla with less than 1% were categorized under the “other” group. Further, an NMDS analysis was used to visualize the differences in beta-diversity. The unweighted unifrac dissimilarity was estimated using the GUniFrac (version 1.8) package (Chen et al., 2021) and used for NMDS analysis. A PERMANOVA analysis was also conducted to test the difference in the microbial beta-diversity across stockpile depths. Additionally, a constrained ordination (using redundancy analysis) was conducted to examine the impact of soil geochemistry on the beta-diversity. The count data (Hellinger transformation) and the geochemical data (normalization) were transformed using the decostand function in vegan (version 2.7.1) *(* Oksanen et al., 2025). Further, a permutation test (permutations = 999) was used to identify the significant edaphic parameters from the geochemical data. These significant geochemical parameters were overlaid on the NMDS output to show their impact on the beta-diversity. A pairwise comparison between the groups was also conducted using the pairwiseAdonis package. Furthermore, a random forest classification model was used using the ranger package (version 0.17.0) to identify the top 10 ASVs explaining the variation across the stockpile depths. The stockpile depth was used as a response, and the ASV feature table was used as the explanatory variable. After running the random forest model, the top 10 ASVs were identified using a permutation test to determine significant (p < 0.05) ASVs. The significant ASVs were then extracted from the count table, and their relative abundances across the stockpile depths depth were investigated.

## Results

### Community composition

**Figure 1:**
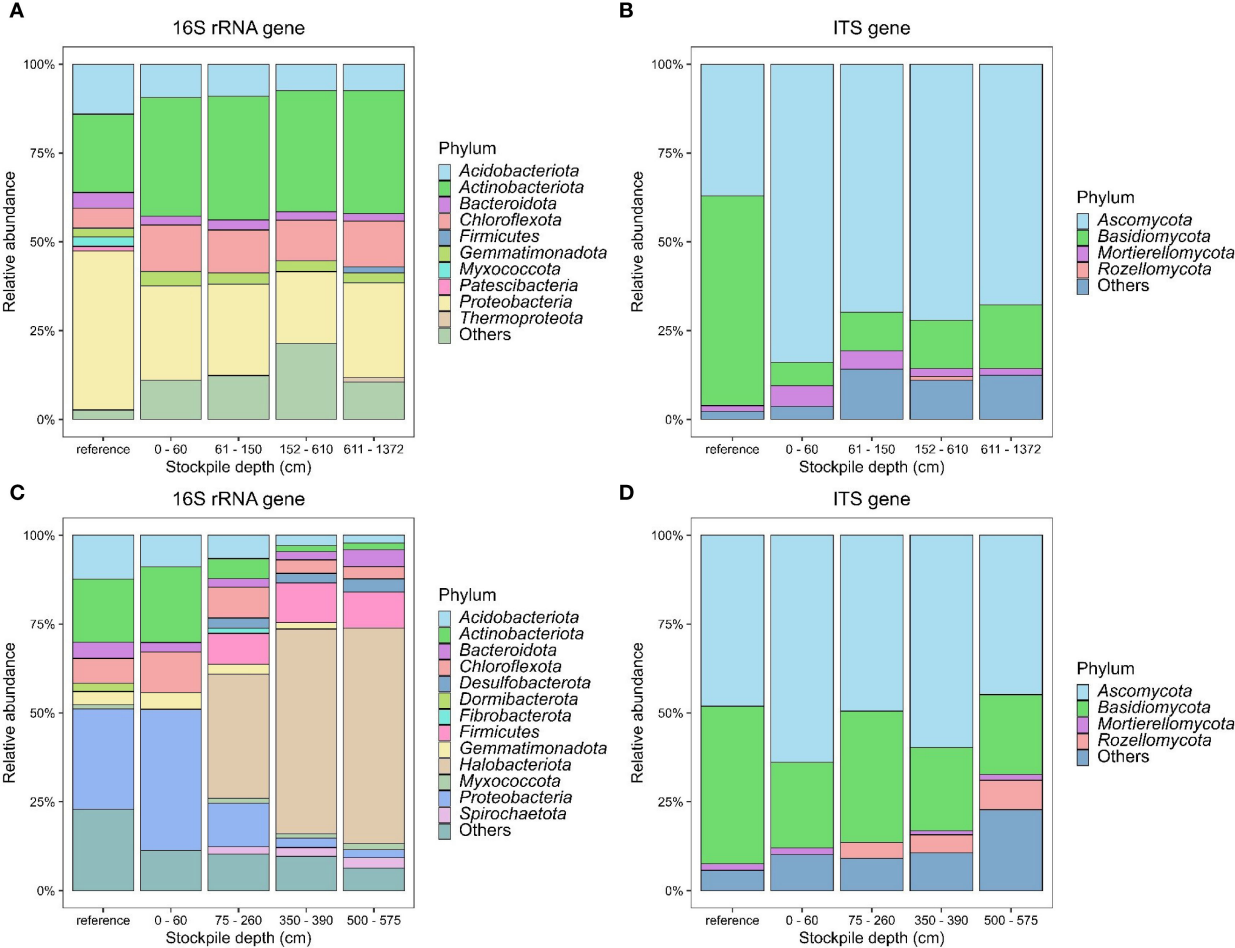
Stacked bar plots showing the relative abundance of dominant 16S and ITS phyla at New Afton **(A, B)** and QR mill **(C, D)**. Unclassified phyla and phyla of abundance lower than 1% were lumped with “others”.

Results showed that the topsoil stockpiles were dominated by the following bacterial phyla: *Acidobacteriota, Actinobacteriota, Bacteroidota, Chloroflexota, Firmicutes, Gemmatimonadota*, and *Proteobacteria* in New Afton (**Figure 1A**) and *Acidobacteriota, Actinobacteriota, Bacteroidota, Chloroflexota, Desulfobacterota, Dormibacterota, Fibrobacterota, Firmicutes, Gemmatimonadota, Halobacteriota, Proteobacteria*, and *Spirochaetota* in QR mill (**Figure 1C**). While the dominant bacterial and archaeal phyla were generally consistent between different stockpile depths (**Figure 1A**) at New Afton, the most significant difference was observed in *Chloroflexota*, which was significantly higher across all depths compared to the reference site (SI-6). Similarly, *Firmicutes*, despite its low relative abundance, was found to be significantly lower in reference compared to other stockpile depths (SI-6). In contrast, considerable differences were observed across all stockpile depths at the QR mill site (**Figure 1C**). The relative abundance of *Acidobacteriota* was lower across all depths compared to the reference (SI-7). Similarly, the relative proportion *Actinobacteriota* decreased gradually with stockpile depth (SI-7). The data also revealed that *Desulfobacterota, Firmicutes*, and *Halobacteriota* increased with stockpile depth (SI-7). In contrast, *Proteobacteria s*howed a decline in relative proportions across stockpile depth (SI-7). *Verrucomicrobiota*, despite its lower proportion, displayed a further reduction in relative abundance with stockpile depth. The most considerable difference in *Proteobacteria* and *Verrucomicrobiota* was observed between the reference and stockpile depths below 60 cm (SI-7).

The dominant soil fungi were mainly composed of *Ascomycota, Basidiomycota, Mortierellomycota*, and *Rozellomycota* at New Afton and QR mill (**Figures 1B, D**). While *Ascomycota* abundance increased with stockpile depths, the relative proportions of *Basidiomycota* decreased at New Afton compared to the reference sites. Interestingly, the proportions of *Glomeromycota* and *Kickxellomycota* at New Afton reduced considerably compared to the reference site (SI-8). In contrast, Ascomycota abundance was consistent across all samples at the QR mill. Basidiomycota abundance reduced slightly at 0–60 cm depth compared to the reference sites.

The random forest model identified the top 10 ASVs from 16S and ITS sequences from New Afton and QR mill that explained the difference in microbial community across the stockpile depths (SI:1–4). At New Afton, ASV_90 was the most important 16S ASV (SI-5). It belonged to the *Gemmatimonadota* phylum. The relative proportion of ASV_90 was significantly lower in the reference sites compared to the stockpiles (SI-1). At QR mill, ASV_603 was the most important 16S ASV (SI-5) and belonged to the *Acidobacteriota* phylum under *Vicinamibacteria* class. Its relative abundance increased in the stockpiles but was not significantly different than the references (SI-2). Among the ITS ASVs, ASV_43 and ASV_126 were the most important sequences for New Afton and QR mill (SI-3 and -4), respectively. ASV_43 increased with stockpile depth compared to the reference sites and was significantly higher at 152–610 cm and 611–1372 cm depth. In contrast, ASV_126 showed a consistent relative proportion across all sites with a minor increase at 0–60 cm depth.

### Alpha diversity

**Figure 2:**
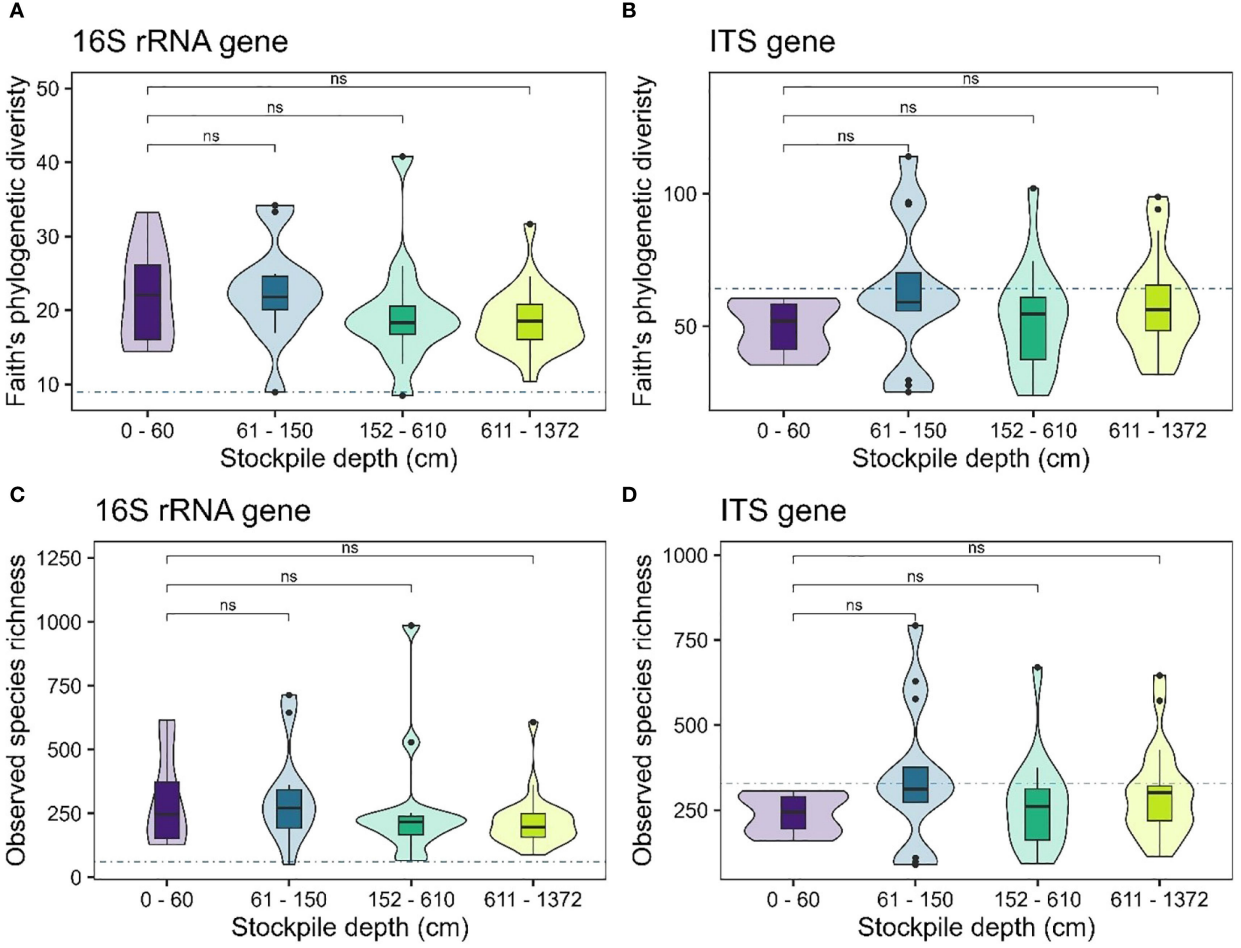
Alpha diversity of 16S and ITS gene across stockpile depths at New Afton. **(A, B)** show the Faith’s PD of New Afton for the 16S rRNA and the ITS gene, respectively. **(C, D)** show the species richness of 16S rRNA and the ITS gene, respectively. The horizontal blue dashed line indicates the mean diversity of the reference sites. The boxes in the boxplot show the interquartile range of the data, with the whiskers indicating the spread of the data, and the dots representing outliers. The violin plot shows the distribution of the data. “ns” denotes no significant differences between the comparisons.

**Figure 3:**
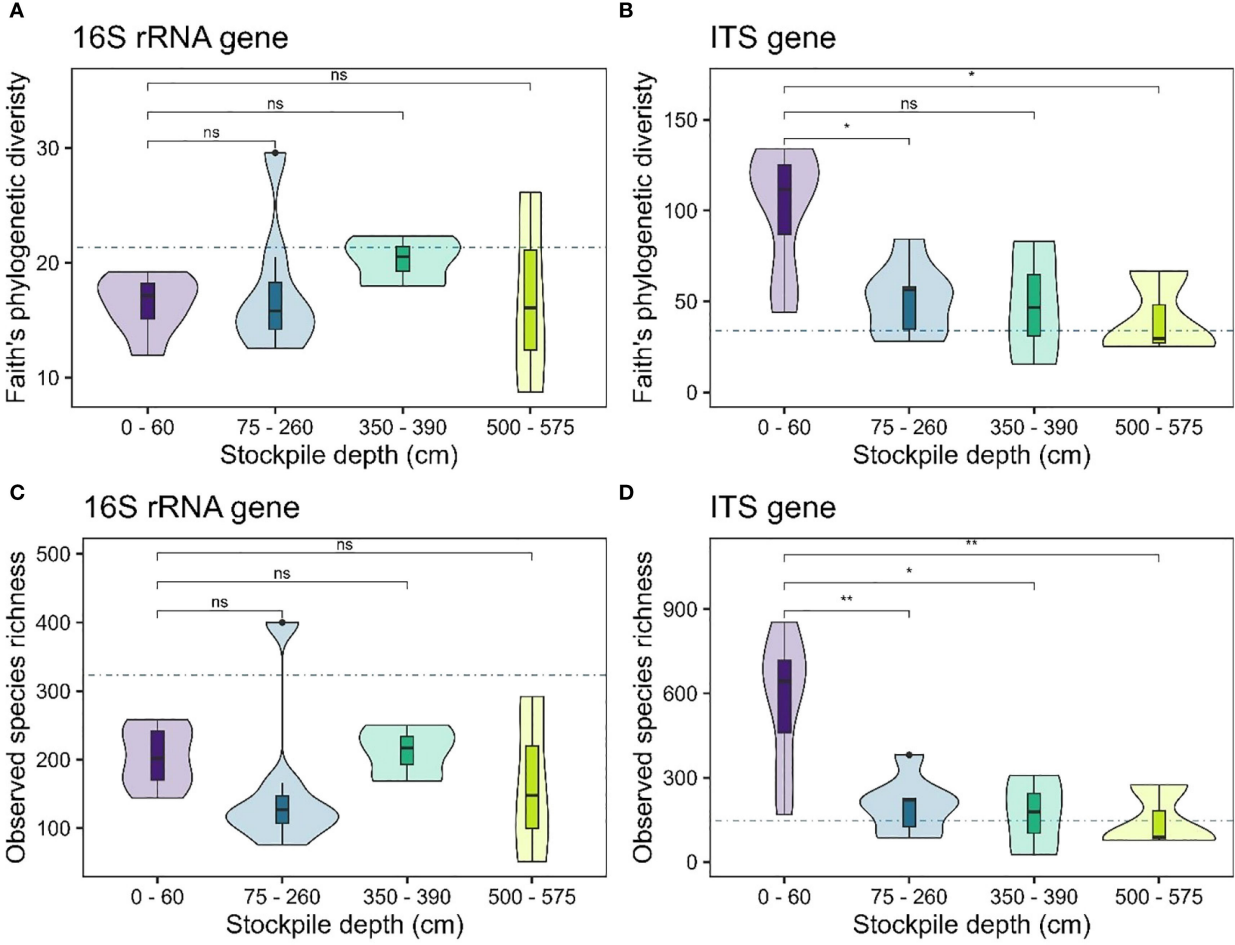
Alpha diversity of 16S and ITS gene across stockpile depths at QR mill. **(A, B)** show the Faith’s PD at QR mill for the 16S rRNA and the ITS gene, respectively. **(C, D)** show the species richness of 16S rRNA and the ITS gene, respectively. The horizontal blue dashed line indicates the mean diversity of the reference sites. The boxes in the boxplot show the interquartile range of the data, with the whiskers indicating the spread of the data, and the dots representing outliers. The violin plot shows the distribution of the data. “ns” denotes no significant differences between the comparisons. “*”, “**” indicate significance at α level 0.05 and 0.01, respectively.

Our results showed no evidence of significant changes in alpha diversity measures for bacterial or fungal communities (observed species richness – **Figures 2A, C**; Faith’s phylogenetic diversity – **Figures 2B, D**) with stockpile depth in New Afton. Interestingly, the reference sites exhibited lower alpha diversity of 16S rRNA genes compared to the stockpiles. For the ITS genes, the reference sites displayed similar alpha diversity compared to the stockpiles (**Figure 2**). At QR mill, the alpha diversity of the 16S rRNA gene showed no significant relationship, indicating that it remained relatively stable across the stockpile depth. The reference site at QR for the 16S rRNA gene displayed slightly elevated levels of alpha diversity (**Figures 3A, C**). In contrast, the alpha diversity (both species richness and Faith’s phylogenetic diversity) shows a significant relationship with stockpile depth. Both species richness and Faith’s phylogenetic diversity were highest at 0–60 cm depths and gradually decreased with stockpile depths (**Figures 3B, D**).

### Beta diversity and geochemical properties

**Figure 4:**
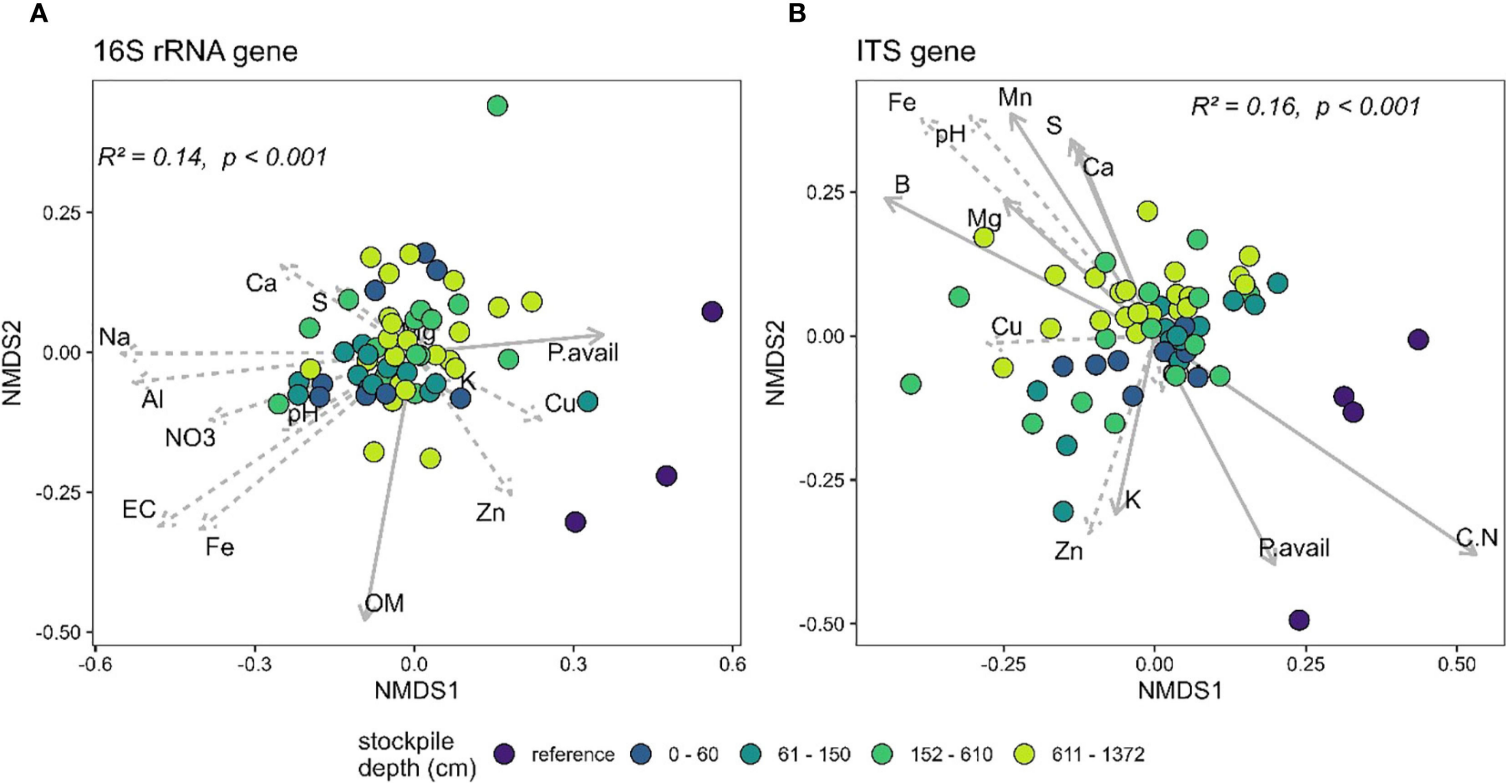
NMDS plot showing the beta-diversity of **(A)** 16S and **(B)** ITS gene at New Afton of reference and different stockpile depths. The NMDS was run on unweighted unifrac dissimilarity. The arrows denote the linear relationship between geochemical parameters and the samples. The arrows with a solid line indicate a significant relationship between the samples (reference site and stockpile depths) and the geochemical parameter, while the dashed arrows indicate a non-significant relationship.

The PERMANOVA analysis of the 16S and the ITS gene sequences at New Afton and QR mill reveals a significant relationship between beta-diversity and stockpile depth. At New Afton, the stockpile depth explained 14% (**Figure 4A**) and 16% (**Figure 4B**) of the variation in 16S and ITS beta-diversity, respectively (**Figure 4**). Additionally, a pairwise comparison between all groups was conducted to examine the differences between all groups (i.e., stockpile depth and references) for both the 16S and the ITS gene. The analysis revealed that the beta-diversity of reference sites was significantly different from samples of all stockpile depths, suggesting that the variation in beta-diversity could primarily be due to the differences between reference sites and stockpiles. Moreover, the reference sites occupied a distinct ordination space with no overlap with any of the stockpile samples. Despite the considerable differences between reference and stockpiles, some significant differences in beta-diversity between stockpile depths were observed. Most significant differences were observed between the topmost layer (e.g., 0–60) and bottommost layers (e.g., 152–610, 611–1372), suggesting significant changes in beta-diversity of 16S and ITS gene at New Afton as the stockpile depth increases. A redundancy analysis was also conducted to understand how geochemical parameters impacted the beta-diversity at New Afton. The results indicate that available P, soil organic matter (OM), and Mg were associated with 16S beta-diversity. The ITS beta-diversity was found to be associated with B, Ca, K, S, available P, NO_3_, C:N ratio, Al, Mg, Mn, and Na. Interestingly, available P influenced both 16S and ITS beta-diversity. Additionally, the available P was found to be associated with the reference sites, suggesting the changes in microbial community structure could be driven by phosphorus availability.

**Figure 5:**
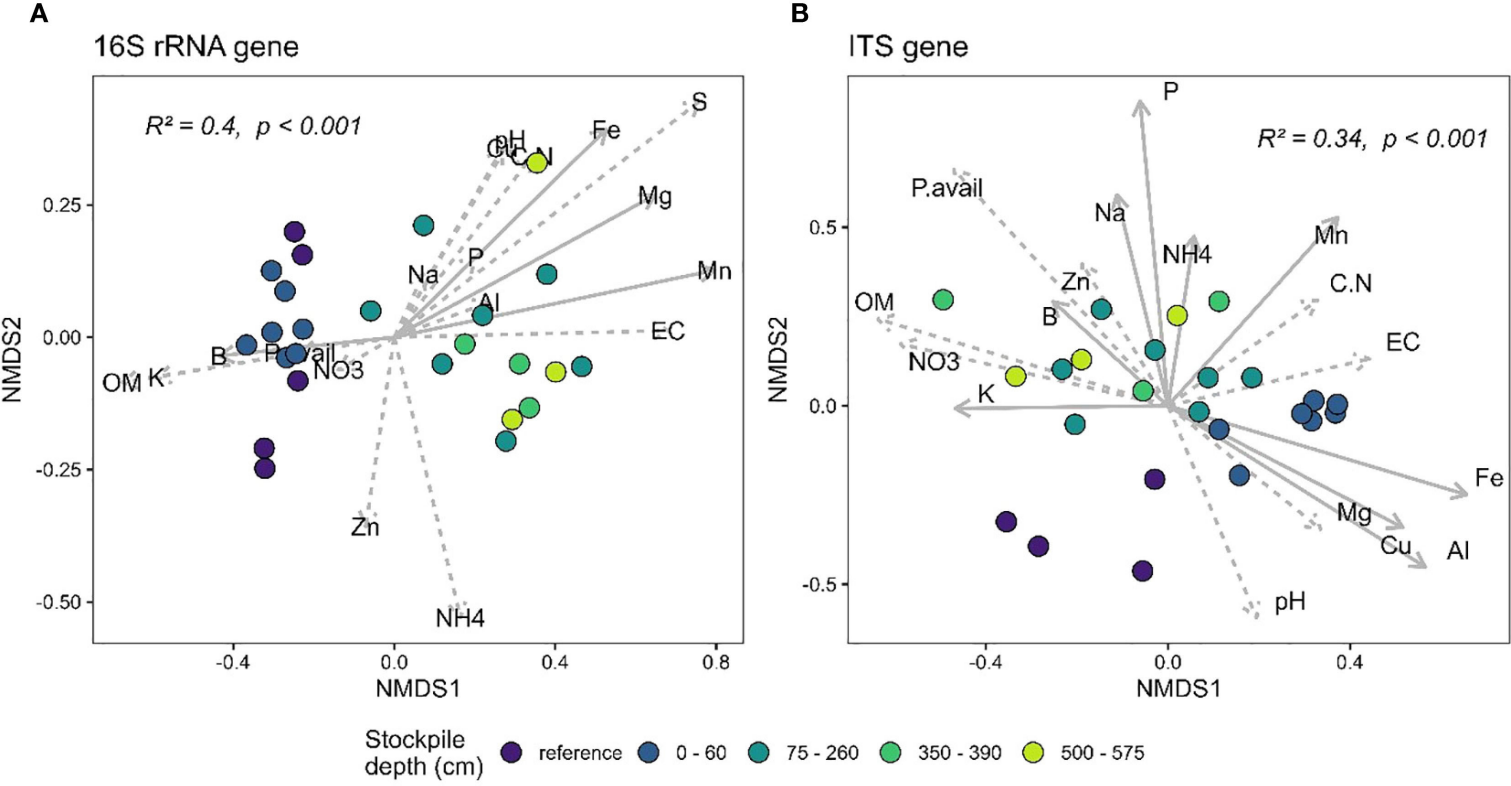
NMDS plot showing the beta-diversity of **(A)** 16S and **(B)** ITS gene at QR mill of reference and different stockpile depths. The NMDS was run on unweighted unifrac dissimilarity. The arrows denote the linear relationship between geochemical parameters and the samples. The arrows with a solid line indicate a significant relationship between the samples (reference site and stockpile depths) and the geochemical parameter, while the dashed arrows indicate a non-significant relationship.

At QR mill, stockpile depth explained 40% (**Figure 5A**) and 34% (**Figure 5B**) of the variation in the 16S rRNA gene and the ITS community. Similar to the results from New Afton, most of the variation could be driven by the difference in community composition between the reference and the stockpile depths. The pairwise PERMANOVA results demonstrate that stockpile samples from all depths are significantly different from the reference sites. However, there were community differences across stockpile depths for 16S and ITS at QR mills. Most of the differences, like New Afton, were observed between the topmost layer (0–60) and the bottommost layers (350–390, 500–575). The redundancy analysis revealed that B, Fe, Mg, and Mn impacted the 16S rRNA beta-diversity. The ITS community was found to be associated with Al, B, Fe, K, Mn, Na, P, Cu, and NH_4_. Interestingly, B was found to be associated with the 16S and ITS communities at both sites (New Afton and QR mill).

## Discussion

On a broad scale, our results are consistent with global microbial surveys (Delgado-Baquerizo et al., 2018; Tedersoo et al., 2022). Similar to Delgado-Baquerizo et al. (2018), our samples were dominated by *Proteobacteria, Acidobacteriota*, and *Actinobacteriota* among the 16S community. Among the ITS community, the samples had a high abundance of *Ascomycota* and *Basidiomycota*, which is similar to the observations made by Tedersoo et al. (2022). However, interesting trends are unraveled upon deeper inspection. At New Afton, the *Chloroflexota* emerged at a higher proportion, particularly in the soil stockpiles. In the reference sites near New Afton, the relative abundance of *Chloroflexota* remained below 10%, while it stayed above 10% for the rest of the stockpiles (SI-6). In some instances, *Chloroflexota’s* relative abundance was found to be over 30% among the stockpile samples (SI-6). The *Chloroflexota* phylum contains microorganisms that exhibit several metabolic pathways to harvest energy, including anoxygenic phototrophy (Pierson and Castenholz, 1974), chemoheterotrophy, and heterotrophy (Freches and Fradinho, 2024). These diverse metabolic pathways for survival and growth potentially allow *Chloroflexota* to thrive in disturbed conditions such as soil stockpiling. Similarly, *Firmicutes’* relative abundance increased with soil depth at New Afton soil stockpiles. A similar observation was made by Bai et al. (2017); Zhang et al. (2017), who found that the relative proportion of *Firmicutes* increased with soil depth (Bai et al., 2017; Zhang et al., 2017). *Firmicutes* employ survival strategies such as endospore formation that enable them to grow under stressful conditions (Fajardo et al., 2019; Filippidou et al., 2016), which is likely the case in the New Afton stockpiles. Among the fungal phyla at New Afton, the relative abundance of *Glomeromycota* reduced with stockpile depth (SI-8). *Glomeromycota* contains arbuscular mycorrhizal fungi that are ubiquitous in soil ecosystems (Brundrett, 1991) and are essential for soil function as they support plant life and benefit biogeochemical cycles (Hawkins et al., 2023; Högberg and Högberg, 2002; Van Der Heijden et al., 2015). A decline in their abundance could severely impact the soil quality of the stockpiles at New Afton.

At QR mill, substantial changes in the 16S rRNA community composition were observed (**Figure 1C**). While the relative abundance of *Acidobacteriota, Actinobacteriota, Verrucomicrobiota*, and *Proteobacteria* declined, the proportion of *Desulfobacterota* and *Halobacteriota* increased with stockpile depth (SI-7). Several studies have reported similar results, where they found that *Acidobacteriota, Actinobacteriota, Verrucomicrobiota*, and *Proteobacteria* decreased with soil depths (Banerjee et al., 2021; Nayak and Mishra, 2020; Zhang et al., 2019; Zhao et al., 2021) likely due to slow growth (Goldman and Green, 2008) or stressful conditions like stockpiles. In contrast, *Desulfobacterota* and *Halobacteriota* proportions increased, likely due to their metabolic preferences (Kuever et al., 2015) and/or cellular ecophysiology (Dundas, 1977). While *Desulfobacterota* respire anaerobically (Thauer et al., 2007), *Halobacteriota* ecophysiology (Dundas, 1977) allow them to sustain disturbances such as soil stockpiling.

Analysis of alpha-diversity of 16S rRNA and ITS gene revealed no significant changes across stockpile depth at New Afton (**Figure 2**). However, the alpha-diversity of the 16S rRNA gene (both SR and PD) of the reference site was substantially lower than the stockpiles (**Figures 2A, C**). A plausible explanation is that soil is regularly added to the stockpiles, which results in frequent disturbances to the ecosystem, while the reference site did not receive such a disturbance. Disturbance is known to sometimes increase the diversity of the ecosystem, which is likely why the 16S rRNA alpha-diversity in stockpiles is higher than the reference sites at New Afton. Like New Afton, 16S rRNA alpha-diversity at QR mill did not show any significant relationship with stockpile depth, but the ITS gene alpha-diversity decreased substantially with depth (**Figures 3B, D**). Other studies have made similar observations, where they found a decline in alpha-diversity with depth (Cabrera-Hernandez et al., 2025; Hernandez et al., 2024). Decrease in microbial diversity with depth has also been observed due to depleted C levels (Eilers et al., 2012; Hansel et al., 2008). In support of this theory, the observed trend of microbial diversity depletion with stockpile depth in QR mill corresponded to the decrease in OM and increase in C:N ratio, especially when the soil was deeper than 60 cm.

The most significant change in diversity at New Afton and QR mill was observed in beta-diversity (**Figures 4**, **5**). Expectedly, the reference sites were significantly different than stockpiles as disturbance is known to impact the beta-diversity (Myers et al., 2015; Vanschoenwinkel et al., 2013). Moreover, stockpiling can substantially change the soil’s abiotic properties (Gupta et al., 2019), which can significantly impact the beta-diversity and ecosystem functioning (Barbosa et al., 2023; Singh et al., 2019a; Xu et al., 2020). Interestingly, soil phosphorus (either available or total), along with Boron, impacted the 16S and ITS beta-diversity for both sites, suggesting their role in determining the community assembly. Phosphorus is a macronutrient and is essential for the survival of all organisms (Butusov and Jernelöv, 2013), and changes in its availability can have a substantial influence on microbial community composition (DeForest and Scott, 2010). Additionally, boron is known to impact not only on microbial community but also their biomass and activity (Vera et al., 2019; Xu et al., 2025). The results also indicate changes in beta-diversity across soil depths, particularly between the uppermost and lowermost depths (ST1 and 2), which is likely due to changes in the geochemistry of the stockpiles (Fischer et al., 2022).

Additionally, a substantial difference between the stockpile at New Afton and the QR mill is observed for both alpha and beta-diversity. The age of the stockpiles could explain this difference between New Afton and QR mills. The stockpile at New Afton is only six years old, while the age of the QR mill stockpile is 20 years. As such, the effects of stockpiling have accumulated for 20 years at the QR mill. Therefore, a significant change in alpha and beta-diversity was observed at the QR mill. Gorzelak et al. (2020) also report a similar observation, where the age of the stockpile changes microbial community composition considerably. It is likely that if the soils at the New Afton site were allowed to stockpile for 20 years, the stockpiles would exhibit similar results.

## Conclusions

Soil stockpiles are a critical resource for the mining industry, as they are later used to reclaim sites at the end of mining operations. Studying soil stockpiling effects through a microbial perspective could provide valuable information about the quality of the soils because microbial communities (key ecosystem engineers) are sensitive to disturbances. Understanding how these disturbances (soil stockpiling) impact microbial communities could allow environmental managers to develop more effective reclamation strategies. To our knowledge, this is the first study that investigated the impact of soil stockpiling at a greater depth (i.e., > 300 cm), which demonstrates that the beta-diversity and in some instances alpha-diversity changes substantially with stockpile depth. The study also shows that stockpiling could potentially impact the quality of soil by reducing the abundance of arbuscular mycorrhizal fungi. Furthermore, the difference in results between the sites can be explained by the age of stockpiling, indicating that the duration of stockpiling has a significant effect on microbial community and must be taken into account for future studies. While this study provides an understanding of microbial community dynamics due to soil stockpiling, future research using transcriptomics and metabolomics can elucidate the functional roles of the microbes in the stockpiles.

## Data Availability

The original contributions presented in the study are publicly available. The processed data, code, and the figures are available on GitHub at https://github.com/jaymicro/Stockpile_microbiome_manuscript.
