# Supplementary material for: Investigating impacts from topsoil stockpile height on soil microbial communities
Source: Front Microbiomes. 2025 Sep 18;4:1607677. doi: 10.3389/frmbi.2025.1607677 (PMC12993518; doi:10.3389/frmbi.2025.1607677)
Supplement: Supplementary file 1 [file SupplementaryFile1.docx]

Investigating Impacts from Topsoil Stockpile Height on Soil Microbial Communities

Ashley Fischer^1^,

Jay P. Singh^1^,

Jonathan Van Hamme^1^,

Eric Bottos^1^,

Lauchlan H. Fraser^1^

^1^Thompson Rivers University, Kamloops, Canada

# Supplementary Information


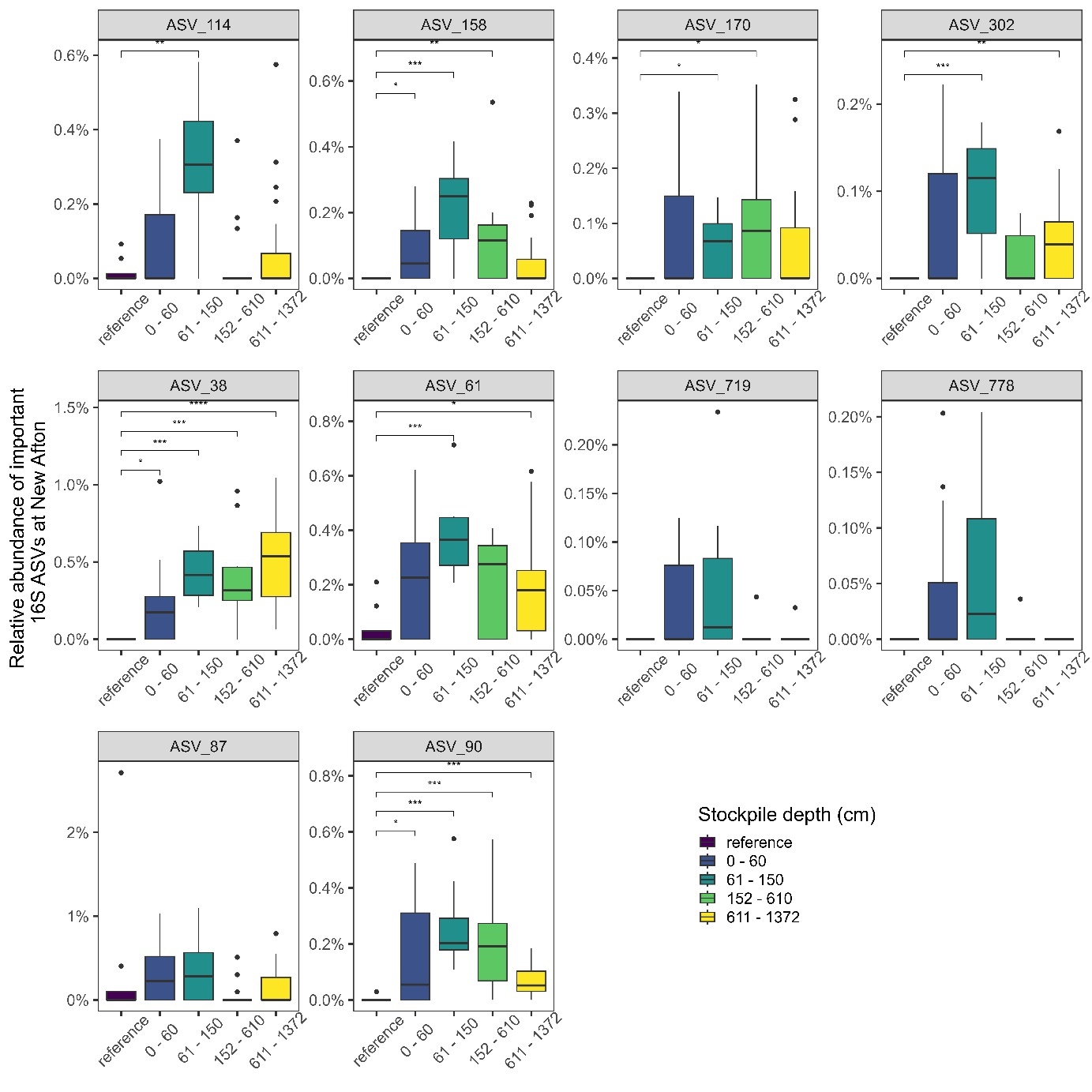


**SI 1:** Relative proportion of the top 10 important 16S ASV at New Afton. These were identified after running a random forest classification model.


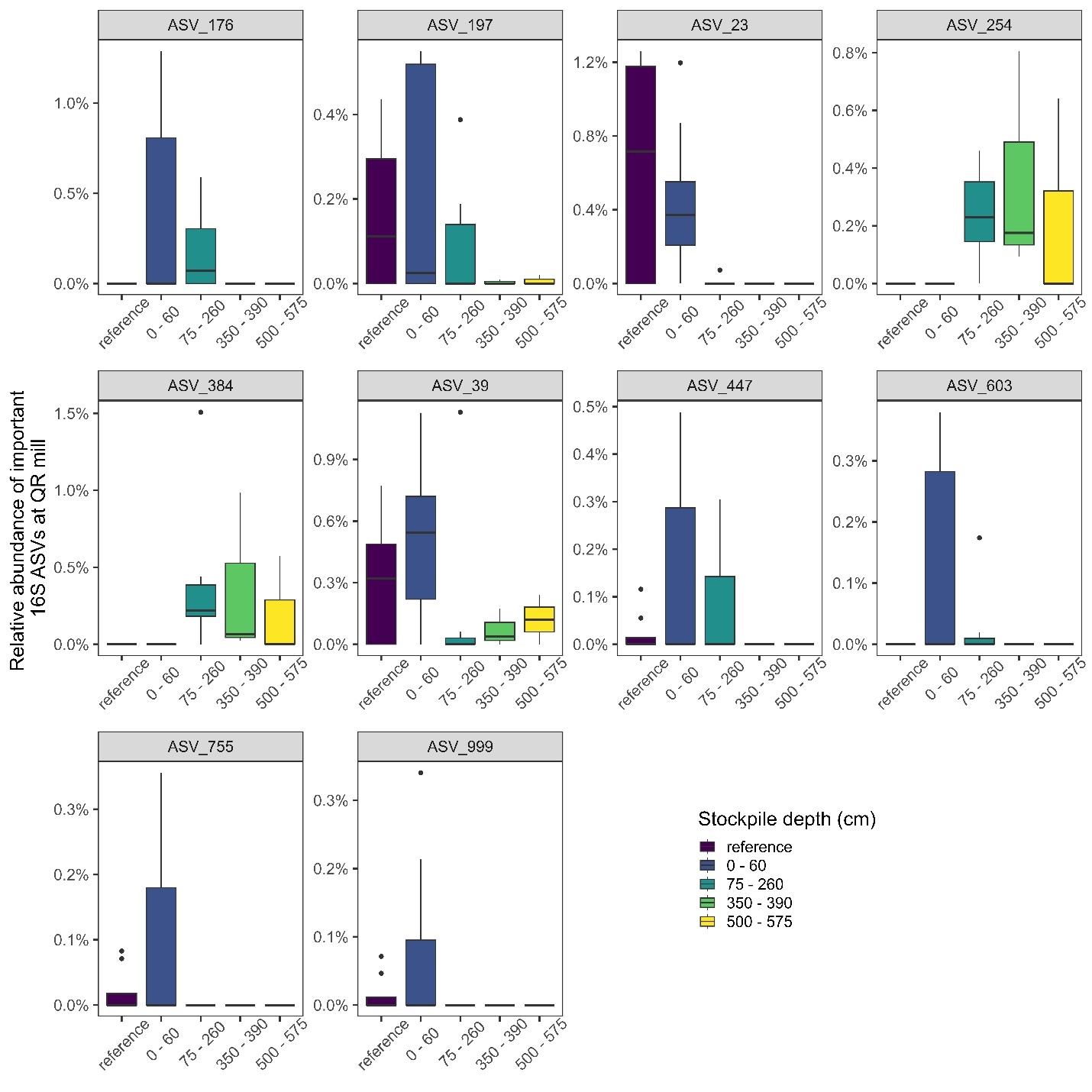


**SI 2:** Relative proportion of the top 10 important 16S ASV at QR mill. These were identified after running a random forest classification model.


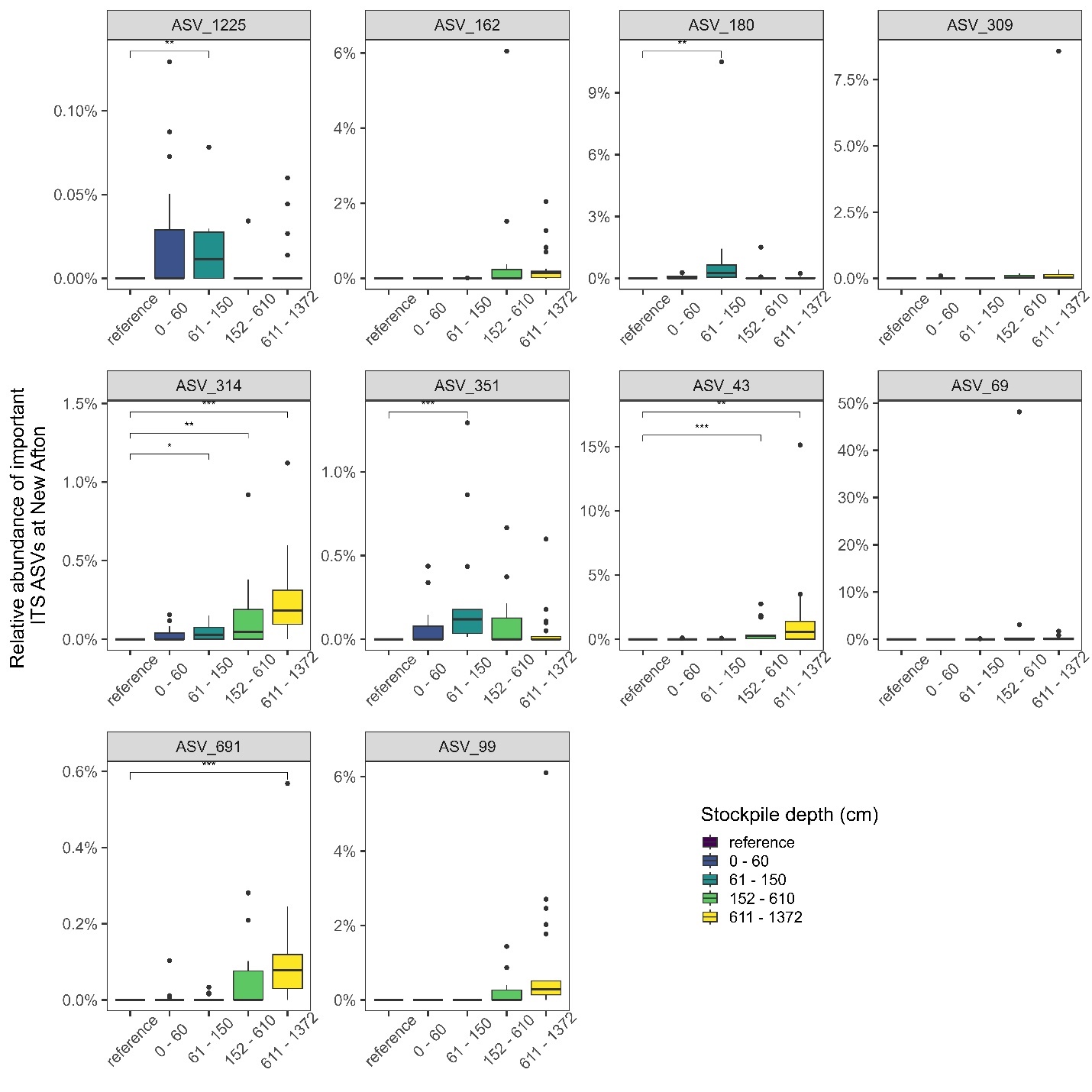


**SI 3:** Relative proportion of the top 10 important ITS ASV at New Afton. These were identified after running a random forest classification model.


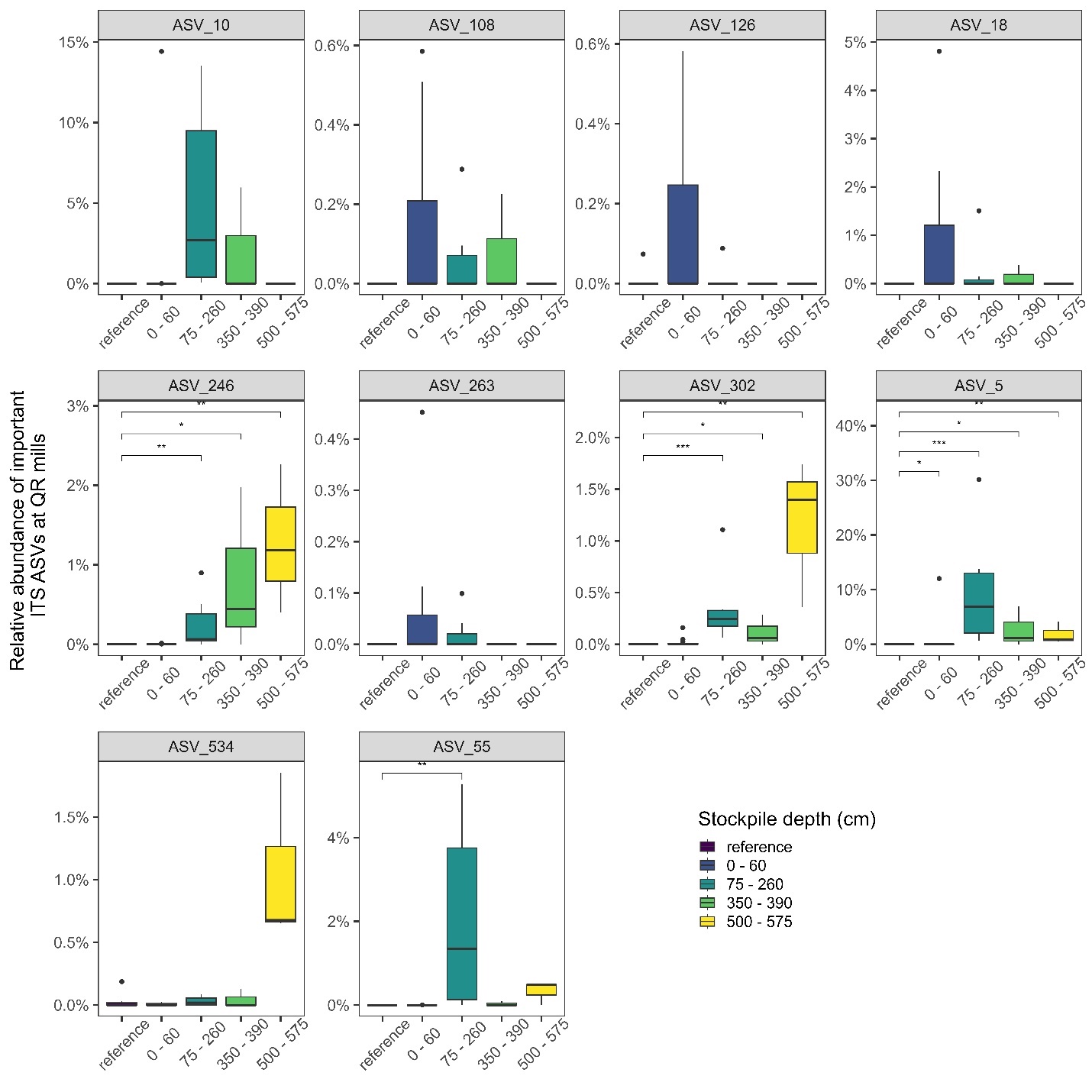


**SI 4:** Relative proportion of the top 10 important ITS ASV at QR mill. These were identified after running a random forest classification model


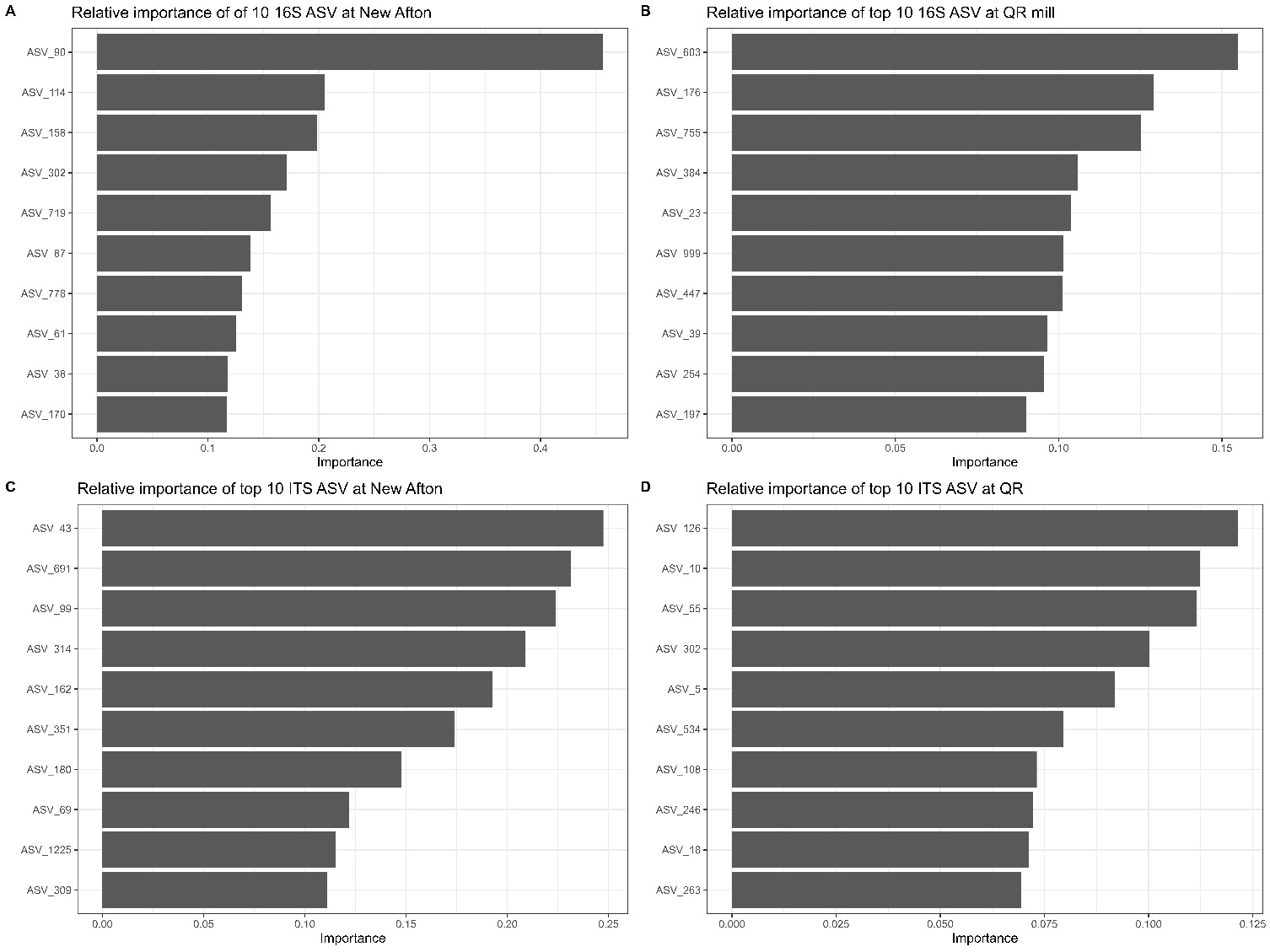


**SI 5:** Relative importance of the top 10 16S ASV identified from (A) New Afton, (B) QR mill, and the top 10 ITS ASV from (C) New Afton, (D) QR mill. The ASVs are sorted by relative importance.


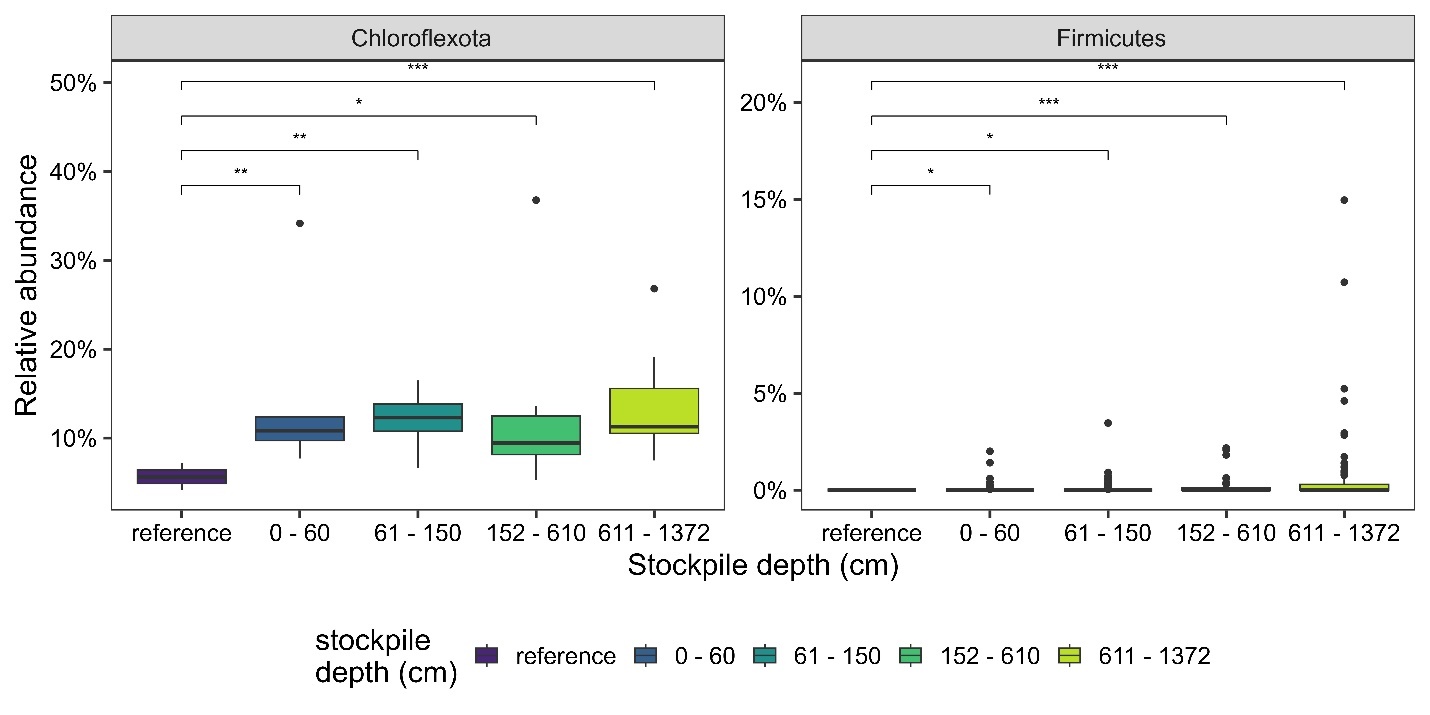


**SI 6:** Relative proportions of Chloroflexota and Firmicutes across stockpile depth in New Afton


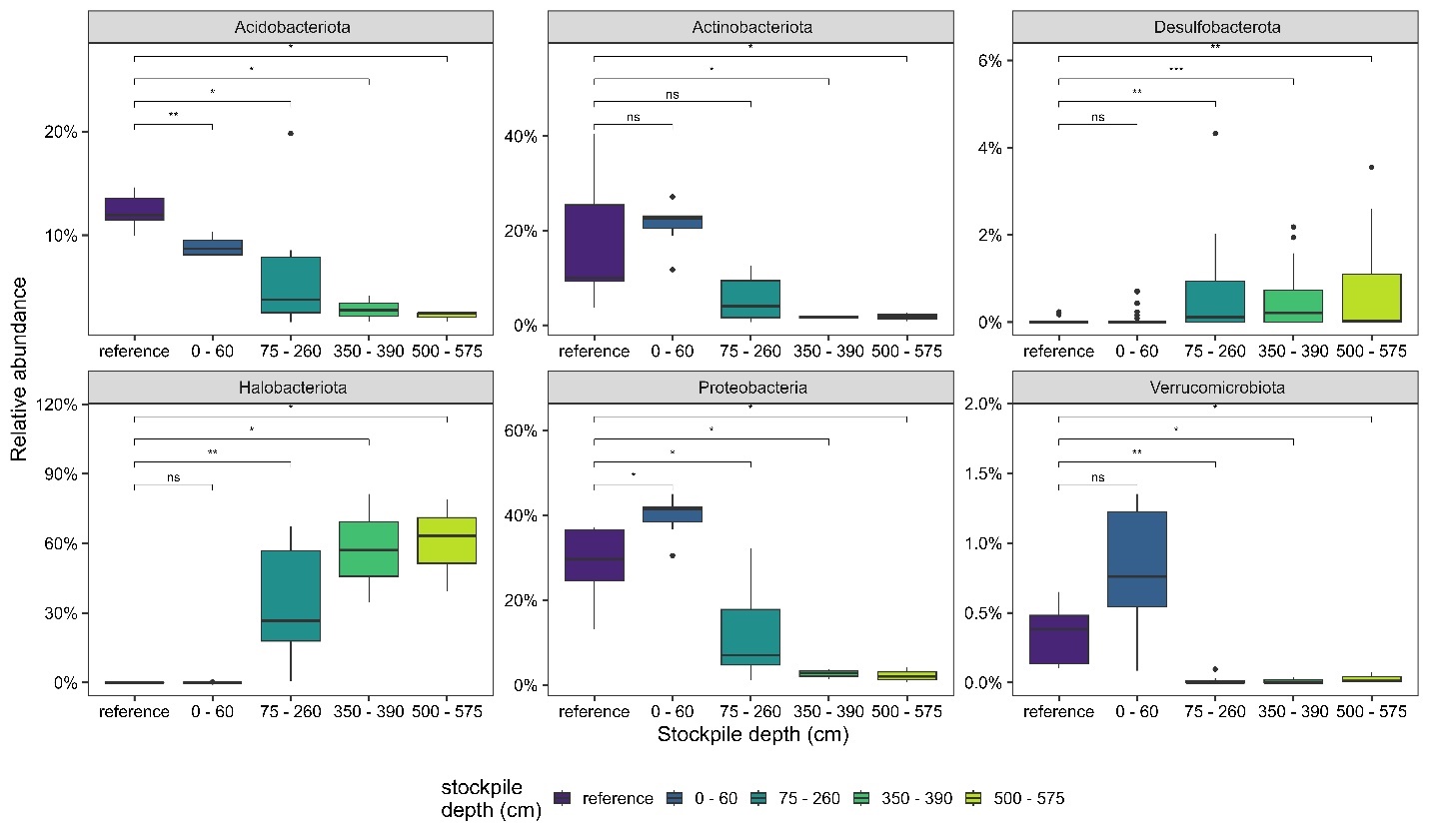


**SI 7:** Relative abundance of Acidobacteriota, Actinobacteriota, Desulfobacterota, Halobacteriota, Proteobacteria, and Verrucomicrobiota, across different stockpile depths


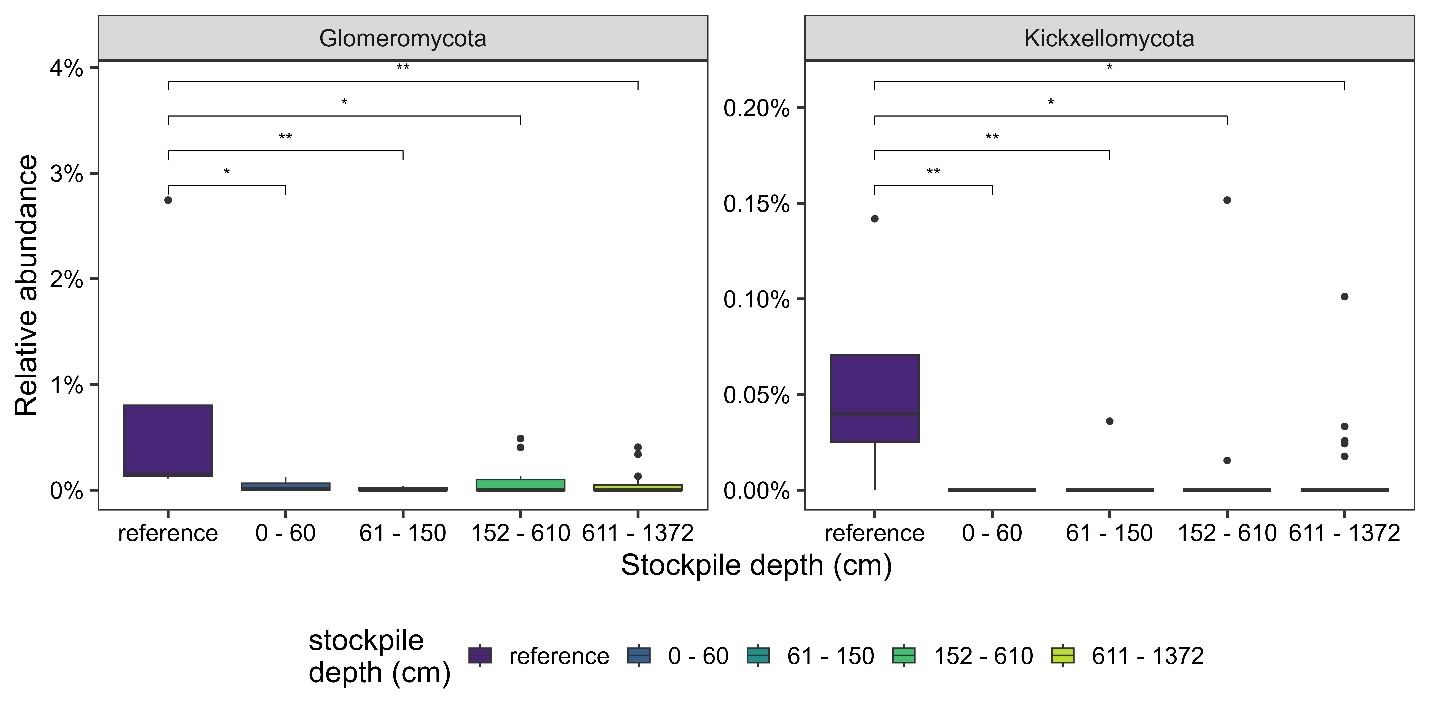


**SI 8:** Relative abundance of Glomeromycota, and Kickxellomycota across different stockpile depths.

**Supplementary Table 1 (ST-1):** Comparison between stockpile depths at New Afton

| New Afton | 16S rRNA | | ITS | |
| --- | --- | --- | --- | --- |
| Comparisons | **F-value** | **p-value** | **F-value** | **p-value** |
| “0 – 60” vs reference | 3.51 | 0.009 ** | 4.62 | 0.006 ** |
| “61 – 150” vs reference | 4.18 | 0.006 ** | 4.95 | 0.001 *** |
| “152 – 610” vs reference | 3.23 | 0.004 ** | 4.26 | 0.001 *** |
| “611 – 1372” vs reference | 4.10 | 0.001 *** | 5.52 | 0.001 *** |
| “0 – 60” vs “61 – 150” | 1.22 | 0.17 | 0.94 | 0.497 |
| “0 – 60” vs “152 – 610” | 1.51 | 0.035 * | 1.35 | 0.041 * |
| “0 – 60” vs “611 – 1372” | 1.97 | 0.003 ** | 2.14 | 0.003 ** |
| “61 – 150” vs “152 – 610” | 1.65 | 0.018 * | 1.72 | 0.005 ** |
| “61 – 150” vs “611 – 1372” | 1.80 | 0.002 ** | 2.75 | 0.001 *** |
| “152 – 610” vs “611 – 1372” | 1.47 | 0.022 * | 1.34 | 0.05 |

**Supplementary Table 2 (ST-2):** Comparison between stockpile depths at QR mill

| QR mill | 16S rRNA | | ITS | |
| --- | --- | --- | --- | --- |
| Comparisons | **F-value** | **p-value** | **F-value** | **p-value** |
| “0 – 60” vs reference | 4.26 | 0.002 ** | 3.96 | 0.002 ** |
| “75 – 260” vs reference | 3.68 | 0.002 ** | 3.33 | 0.002 ** |
| “350 – 390” vs reference | 3.32 | 0.025 * | 2.13 | 0.061 |
| “500 – 575” vs reference | 3.26 | 0.021 * | 2.83 | 0.05 * |
| “0 – 60” vs “75 – 260” | 5.53 | 0.003 ** | 2.98 | 0.005 ** |
| “0 – 60” vs “350 – 390” | 6.74 | 0.009 ** | 2.30 | 0.002 ** |
| “0 – 60” vs “500 – 575” | 6.61 | 0.01 ** | 3.52 | 0.011 * |
| “75 – 260” vs “350 – 390” | 0.81 | 0.699 | 0.93 | 0.53 |
| “75 – 260” vs “500 – 575” | 0.94 | 0.50 | 1.40 | 0.11 |
| “350 – 390” vs “500 – 575” | 0.40 | 0.9 | 0.99 | 0.5 |

**16S rRNA raw reads stats**

| **Sample_id** | **16S rRNA Raw reads** |
| --- | --- |
| 1-1 | 118605 |
| 1-10 | 170180 |
| 1-15 | 230018 |
| 1-2 | 507463 |
| 1-20 | 8399 |
| 1-25 | 212769 |
| 1-3 | 204452 |
| 1-30 | 119170 |
| 1-35 | 132282 |
| 1-4 | 294336 |
| 1-40 | 236617 |
| 1-45 | 64935 |
| 1-5 | 83951 |
| 2-1 | 149085 |
| 2-10 | 205397 |
| 2-15 | 391946 |
| 2-2 | 193510 |
| 2-20 | 205785 |
| 2-25 | 363598 |
| 2-3 | 379121 |
| 2-30 | 349279 |
| 2-35D | 92045 |
| 2-4 | 159672 |
| 2-5 | 505056 |
| 3-1 | 127956 |
| 3-20 | 130942 |
| 3-3 | 193036 |
| 4-4 | 360961 |
| 4-45 | 350811 |
| 4-5 | 290610 |
| Forest | 43679 |
| NA-R2 | 31141 |
| NA-R3 | 4210 |
| NA.R1 | 377474 |
| NA.R2 | 319074 |
| NA.R3 | 267666 |
| QR-R1 | 1944 |
| QR-R2 | 9421 |
| QR-R3 | 11747 |
| **Sample_id** | **16S rRNA Raw reads** |
| QR.R1 | 331920 |
| QR.R2 | 263 |
| QR.R3 | 54 |
| QR1.10 | 21727 |
| QR1.105 | 76883 |
| QR1.20 | 1980466 |
| QR1.200 | 763993 |
| QR1.350 | 445330 |
| QR1.500 | 173416 |
| QR1.75 | 582511 |
| QR2.10 | 1781991 |
| QR2.108 | 82783 |
| QR2.20 | 1739023 |
| QR2.250 | 39689 |
| QR2.365 | 73515 |
| QR2.380 | 20273 |
| QR2.550 | 34827 |
| QR2.60 | 1083364 |
| QR3.10 | 1285901 |
| QR3.20 | 2811400 |
| QR3.210 | 64876 |
| QR3.565 | 52363 |
| QR3.90 | 204779 |
| SC1-00.3a | 497 |
| SC1-00.6a | 325 |
| SC1-00.9a | 368 |
| SC1-01.2a | 26 |
| SC1-01.5a | 189 |
| SC1-04.6a | 85 |
| SC1-07.6a | 720 |
| SC1-09.1a | 460 |
| SC1-10.7a | 5 |
| SC1-12.2a | 140 |
| SC1-13.7a | 79 |
| SC1-3.00a | 47 |
| SC2-00.3a | 338 |
| SC2-00.6a | 750 |
| SC2-00.9a | 91 |
| SC2-01.2a | 407 |
| **Sample_id** | **16S rRNA Raw reads** |
| SC2-01.5a | 140 |
| SC2-04.6a | 360 |
| SC2-06.1a | 153 |
| SC2-07.6a | 38 |
| SC2-09.1a | 104 |
| SC2-10.7a | 149341 |
| SC2-10.7d | 567 |
| SC2-12.2a | 148930 |
| SC2-13.7a | 97837 |
| SC2-3.00a | 75 |
| SC3-00.3a | 133 |
| SC3-00.6a | 86769 |
| SC3-00.9a | 7252 |
| SC3-01.2a | 62897 |
| SC3-01.5a | 154101 |
| SC3-04.6a | 118069 |
| SC3-06.1a | 312 |
| SC3-06.1c | 121902 |
| SC3-07.6a | 80218 |
| SC3-09.1a | 156172 |
| SC3-10.7a | 42054 |
| SC3-12.2a | 118173 |
| SC3-13.7a | 143494 |
| SC3-15.2a | 92618 |
| SC3-3.0a | 214402 |
| SC4-00.3a | 81094 |
| SC4-00.6a | 172964 |
| SC4-00.9a | 102984 |
| SC4-01.2a | 1575 |
| SC4-01.5a | 5119 |
| SC4-04.6a | 145064 |
| SC4-06.1a | 55523 |
| SC4-07.6a | 63646 |
| SC4-09.1a | 170873 |
| SC4-10.7a | 157517 |
| SC4-12.2a | 345158 |
| SC4-13.7a | 297 |
| SC4-15.2a | 131786 |
| SC4-3.00a | 172817 |

**ITS raw reads stats**

| **Sample_id** | **ITS raw reads** |
| --- | --- |
| 1-10 | 181100 |
| 2-15 | 483823 |
| 2-20 | 155070 |
| 2-3 | 162311 |
| 2-5 | 201464 |
| 3-3 | 162632 |
| 4-25 | 169033 |
| FOREST | 1363 |
| GRASSLAND | 20624 |
| NA-R2 | 3750 |
| NA-R3 | 14014 |
| QR-R1 | 33453 |
| QR-R2 | 232590 |
| QR-R3 | 257527 |
| QR.R1 | 157611 |
| QR.R2 | 26753 |
| QR.R3 | 93 |
| QR1-10 | 70323 |
| QR1-105 | 39386 |
| QR1-20 | 97456 |
| QR1-200 | 24552 |
| QR1-350 | 73975 |
| QR1-500 | 56840 |
| QR1-75 | 46901 |
| QR2 | 2263 |
| QR2-10 | 48925 |
| QR2-108 | 65920 |
| QR2-20 | 151306 |
| QR2-250 | 53834 |
| QR2-380 | 613404 |
| QR2-550 | 34433 |
| QR2-60 | 110138 |
| QR3 | 2415 |
| QR3-10 | 80540 |
| QR3-20 | 144189 |
| QR3-200 | 381100 |
| QR3-210 | 975 |
| QR3-365 | 55111 |
| QR3-565 | 231092 |
| QR3-90 | 50563 |
|  |  |
| **Sample_id** | **ITS raw reads** |
| SC1-10 | 70426 |
| SC1-15 | 70924 |
| SC1-2 | 47418 |
| SC1-20 | 27220 |
| SC1-25 | 51358 |
| SC1-3 | 58367 |
| SC1-30 | 101150 |
| SC1-35 | 90031 |
| SC1-4 | 122571 |
| SC1-40 | 195548 |
| SC1-45 | 112486 |
| SC1-5 | 103366 |
| SC2-1 | 53322 |
| SC2-10 | 162974 |
| SC2-2 | 68343 |
| SC2-25 | 96901 |
| SC2-30 | 38730 |
| SC2-35A | 102566 |
| SC2-35D | 65571 |
| SC2-4 | 173820 |
| SC2-40 | 38741 |
| SC2-45 | 113430 |
| SC3-1 | 98458 |
| SC3-10 | 111490 |
| SC3-15 | 143296 |
| SC3-2 | 148940 |
| SC3-20 | 139838 |
| SC3-25 | 55793 |
| SC3-25star | 100121 |
| SC3-3 | 99224 |
| SC3-30 | 110141 |
| SC3-35 | 58495 |
| SC3-4 | 85974 |
| SC3-40 | 56091 |
| SC3-45 | 67462 |
| SC3-5 | 163153 |
| SC3-50 | 33024 |
| SC4-1 | 59626 |
| SC4-10 | 56388 |
| SC4-15 | 57377 |
|  |  |
| **Sample_id** | **ITS raw reads** |
| SC4-2 | 98237 |
| SC4-20 | 11700 |
| SC4-25 | 647 |
| SC4-3 | 48237 |
| SC4-30 | 82345 |
| SC4-35 | 60769 |
| SC4-4 | 81893 |
| SC4-40 | 119847 |
| SC4-45 | 64967 |
| SC4-5 | 68456 |
| SC4-50 | 111800 |
